# Policy implications for halting human trafficking and sexual exploitation at U.S. colleges and universities: results from a 12-campus survey

**DOI:** 10.3389/fpsyt.2026.1773818

**Published:** 2026-04-20

**Authors:** Lianne A. Urada, Ashley Weitensteiner

**Affiliations:** 1 Department of Medicine, University of California, San Diego, San Diego, CA, United States; 2 School of Social Work, San Diego State University, San Diego, CA, United States

**Keywords:** human trafficking, postsecondary education, psychological harm, sex exchange, sex trafficking, sexual assault, sexual exploitation, universities and colleges

## Abstract

Human trafficking is a priority concern in the U.S, but few research studies have investigated the nature and extent of its occurrence on U.S. college campuses nor strategies to stop it. The objective of this research was to identify its occurrence (force/coercion/fraud into sex exchanges for something of monetary or other value) among college students in San Diego County and Imperial Valley, California, where some of the highest documented rates of human trafficking occur nationally, and its implications for policy. Methods: College students (n = 971) from 12 campuses in Southern California responded to fliers posted on their campuses by completing online self-administered surveys. Results: Nearly one in five students (18%) surveyed reported experiencing human trafficking in college. In bivariate analyses, trafficked students were more likely to be BIPOC, LGBTQ, foster youth, fraternity/sorority members, use illicit substances, experience abuse (physical, emotional, sexual, or being told to recruit others), transport/sell drugs, perform labor, and exchange sex for grades/schoolwork. Multiple logistic regression analyses (adjusting for age, sexual orientation, race) revealed those trafficked were more likely as college students to exchange sex across the U.S.-Mexico border (OR = 4.02; CI: 2.52-6.17), be inhibited to seek academic counseling (OR = 2.58; CI = 1.63-4.10), acquire a sexually transmitted infection (OR:1.63; CI: 1.04-2.57), wonder how they would afford their next meal (OR:1.20; CI: 1.03-1.40), and feel pressure from others (i.e., instructors, peers) to engage in sex (OR:2.69; CI:11.75-4.12). Conclusions: Strategies may need to: 1) expand Title IX implementation to encompass human trafficking/sexual exploitation information at all universities, and 2) amend or introduce state laws mandating human trafficking prevention awareness training at schools to include colleges/universities.

## Introduction

Human trafficking is a priority concern in the U.S (1). The U.S. national human trafficking hotline identified 31,764 victims of human trafficking since 2007 (2). However, few research studies have investigated the nature and extent of its occurrence on U.S. college campuses, and its implications for policy, until now. The U.S. defines human trafficking as “force, fraud, or coercion” into commercial sex (or other services) (3). Anyone under age 18 engaged in commercial sex is considered trafficked because they cannot legally consent to it (3). Since its landmark legislation, the Victims of Trafficking and Violence Protection Act of 2000, guides nations’ scores on how well they address human trafficking (3). However, 25 years later, after multiple iterations, legal definitions confer automatic human trafficking status only on youth engaged in commercial sex under age 18. Yet transitional age youth over age 18 face similar conditions and risks for exploitation during their transition to adulthood. This study is one of the first to focus on the sexual exploitation aspect of human trafficking, or “the exchange of sex for something of monetary or other value” under coercion, force, or fraud as experienced by U.S. college students.

Although there is a paucity of human trafficking information among U.S. college and university students, studies on adolescents and high school youth have shown that trafficking of minors is higher in the general population than generally believed, including in San Diego, California (4–7). A national sample captured minors’ exchange of sex for drugs, money, food, shelter, and other favors in the general population, for example, including those 18–27 years of age (8). Several websites also describe the urgent issue of the trafficking of college students. The U.S. Department of Homeland Security designed a “Blue Campaign Human Trafficking Awareness Guide for Student Leaders on College Campuses” (9).

Science tells us that the executive functioning of the human brain continues to develop through age 25 or even 30 (10). Therefore, arguably, the transition to adulthood could be the riskiest time to be trafficked. Furthermore, the transition into college often involves stepping away from familiar social networks and seeking new connections, which can leave students vulnerable to manipulation. A recent longitudinal study following high school students as they transitioned to college found that more than half of the students (57%) reported increased loneliness once in college (11). The lack of social connectedness may present as a vulnerability to traffickers who seek to first befriend potential victims. Financial instability is also typical as many students take out loans and incur debt to attend school, further increasing their susceptibility, and in many cases, leading to homelessness (e.g., living out of their cars), and food insecurity (12, 13). Additionally, traffickers may take advantage of the culture of substance use experimentation that exists on many campuses to exploit and exert control over students. The lure of alternative ways to make money and support themselves, especially those with little family support, makes college-age students even more vulnerable to sexual exploitation, leading to human trafficking.

In 2017, California enacted the Human Trafficking Prevention Education and Training Act to combat trafficking in middle and high schools through educational campaigns and training for students and teachers (14); other states followed suit. Experts now understand that college students, too, are vulnerable to such predators. The U.S. Department of Homeland Security’s Blue Campaign began distributing guidance to campus police about human trafficking only after college personnel alerted them about the campus crime (15). However, there is a lack of data on the extent that human trafficking occurs among college students and what factors may lead to their increased vulnerability. As a result, human trafficking prevention training has not been uniformly addressed at universities.

Therefore, the objective of this research was 1) to identify the nature and extent of human trafficking and sexual exploitation occurring among college students in two southern California regions: San Diego County and Imperial Valley, U.S.-Mexico border regions with some of the highest documented rates of human trafficking nationally (16); and 2) to determine the characteristics significantly associated with being trafficked as a college student in order to inform policies that could stop it from happening in the first place or to identify and intervene in cases of continuing exploitation.

Human trafficking vulnerability is hypothesized in this paper to be associated with social and structural factors such as being an undergraduate experiencing financial insecurity, being a former or current foster youth, BIPOC (Black, Indigenous, People of Color), and LGBT (Lesbian, Gay, Bisexual, Transgender). Second, students forced/coerced into commercial sex as college students are hypothesized to report worse mental health outcomes, including lower help-seeking behaviors, than those who were not trafficked. Therefore, this study has the potential to have a large impact in higher education policies on human trafficking prevention and response, and to fill the gap in our understanding of the nature and extent of human trafficking on U.S. college and university campuses.

## Methods setting

In 2024, the U.S. national human trafficking hotline identified 3,603 California victims (2). Studies in the U.S.-Mexico border region, including minors, have identified sex trafficking in the region (17, 18). San Diego County shares a length of border with Mexico where cross-border human trafficking and smuggling is a unique geopolitical feature of the area. Similarly, Imperial Valley, where a large majority of Hispanic/Latine of Mexican origin reside, borders Baja California (in the cities of Calexico and Mexicali).

### Design

We applied a multi-site, cross-sectional, self-administered survey design to examine experiences of human trafficking and sexual exploitation among college students in San Diego County and Imperial Valley (located in southern California). Students were recruited from 12 different universities and colleges, including four-year public and private universities and community colleges within San Diego and Imperial Counties in California. In addition, students reported transferring from colleges/universities in other California cities (e.g., Los Angeles, Riverside, San Bernardino, and northern California) and neighboring states (i.e., Arizona).

Study approval was obtained from the institutional review boards of San Diego State University (IRB#: HS-2022-0162), San Diego Community College District, Grossmont-Cuyamaca Community College District, and Southwestern College. Six other major public and private universities/colleges in the region also allowed study flyers to be posted on their campuses (2022-2023).

Due to the sensitive nature of the study, care was taken to obtain informed consent after participants were made aware of the study’s study purpose, design, and anonymous nature of the survey. The voluntary nature of participation was emphasized, and at several points in the survey, participants were reminded they could exit the survey at any time. A prompt also provided them the option to share their contact information if they wished to speak with someone (licensed professional or human trafficking survivor) within 48 hours for a direct connection to human trafficking and or mental health resources. A resource list including mental health and human trafficking specific services, located both on and off campus and tailored to each college or university, was provided at the beginning and end of the survey. Participants who declined to consent also received the list of resources. Importantly, to ensure survey responses were completely anonymous, participants provided their email address only to receive a $15 Amazon gift card for their participation in the study via a separate linked form, keeping their identity separate from their survey responses.

### Recruitment

The 12-campus study represented a majority of universities in San Diego and Imperial Valley. Students were recruited via targeted placement of study flyers on college campuses in areas frequented by students and approved by the school and/or IRB. These areas included bathroom stalls, campus bulletin boards, hallways, student centers, and libraries. The research teams posted approximately 100 flyers on each campus, trying to reach and restrict responses to 100 for each college/university to ensure representation. While some participants likely shared the survey with others, we did not track this activity due to participant anonymity. Although all students had an equal chance to respond to the flyer, not all students may have seen or responded to the flyers. Therefore, the sample did not represent all students on every campus.

Flyers described the study and included a QR code and survey link that directed students to the online survey, hosted on a Qualtrics survey platform. Specifically, the flyer said the purpose of the research study was to “explore the nature and extent of sexual exploitation among college students in San Diego County and Imperial Valley and any links to food insecurity, housing instability, and stigma faced as a result.” The flyer said the survey will include questions surrounding sex trade/sexual exploitation, trafficking, gender norms, and stigma. Informed by an advisory board of human trafficking survivor leaders, the fliers intentionally avoided terms like “human trafficking” or “modern slavery.” Students may not immediately identify their experiences as human trafficking. Therefore, the term “sexual exploitation” was used on the flyer instead, inviting those to participate if they were interested in participating in a study about sexual exploitation among college students and may have knowledge about these topics and opinions unique to their background.

To be eligible to participate in the study, participants were 1) 18 years or older, and 2) currently enrolled students or recent alumni of one of the participating institutions. They did not need personal experience with sexual exploitation to complete the survey but needed to have opinions or knowledge about these topics unique to their background. Participants completed the survey on their own electronic device at a time and place of their choosing. Surveys took approximately 15 to 45 minutes to complete.

### Study development, measures, and analyses

The survey instrument was developed in collaboration with a human trafficking survivor leader advisory group. Our main outcome measure of human trafficking was defined as experiencing force, fraud, or coercion into selling sex (exchanging for money or something of monetary or other value), aligning with U.S. federal definitions (2). We made a distinction in the survey between human trafficking and exchanging sex for something of monetary or other value free of coercion, force, or fraud. The survey asked about both separately. In this paper, we focus on the human trafficking variable as the outcome of interest.

The survey included questions about selling sex, sexual exploitation, human trafficking (sex and labor trafficking), gender norms, and stigma. It also included questions about attitudes related to human trafficking, motives, and circumstances around selling sex, such as financial, food, and housing insecurity. These questions were informed in consultation with human trafficking survivors and by previous survey research with university students mostly outside of the U.S (19, 20). Additional domains included sociodemographic characteristics, substance use, mental health, and help-seeking behaviors. For example, 10 of 22 items were adapted from the Impact of Event Scale (IES-R) (21) and four items from the Psychological Sense of School Membership scale (PSSM) (22). While most questions asked about the survey respondent’s direct experience, some questions allowed them to answer questions about someone they knew as a college student. For example, the question asked “If you or a friend/family member exchanged a sexual act for something of monetary or other value, what were the reasons for doing so?”.

For bivariate analyses presented in **Table 1**, categorical variables were compared by trafficking experience using Pearson chi-square tests of independence. Ordinal variables, including food insecurity and financial stability, were analyzed as categorical variables in chi-square tests. For items allowing multiple responses (e.g., race/ethnicity, barriers to help-seeking, and recommended campus response), the categories for each response option were recoded as separate binary variables and analyzed independently. Variables that were conditional on a prior response (e.g., the source of pressure reported among students who reported sexual pressure at college) were restricted to relevant subsamples, as noted in **Table 1**. Age was analyzed as a continuous variable using independent samples t-tests.

**Table 1:** Socio-demographic characteristics of those trafficked vs. not trafficked as a college student among those surveyed across 12 San Diego/Imperial Valley colleges/universities, bivariate analyses* n=971.

Variable	Total n (%) = 971	Not Trafficked n (%) = 799 (82.3)	Trafficked n (%) = 172 (17.7)	P-value
Socio-demographics				
**Year in school**				**0.001**
First year	230 (24.2)	193 (24.2)	37 (21.5)	
Second year or greater	587 (61.9)	491 (61.5)	96 (55.8)	
Graduate	97 (10.0)	63 (7.9)	34 (19.8)	
Recently graduated (alumni)	35 (3.7)	30 (3.8)	5 (2.9)	
**Race**				
American Indian or Alaska Native	46 (4.7)	33 (4.1)	13 (7.6)	0.055
Asian/Pacific Islander	176 (18.1)	152 (19.0)	24 (14.0)	0.117
Black/African American	166 (17.1)	115 (14.4)	51 (29.7)	**0.001**
White	373 (38.4)	312 (39.0)	61 (35.5)	0.381
Latine	348 (35.8)	302 (37.8	46 (26.7)	**0.006**
Other	22 (2.3)	20 (2.5)	2 (1.2)	0.284
**Gender**				**0.001**
Woman	669 (68.9)	576 (72.1)	93 (54.1)	
Man	242 (24.9)	180 (22.5)	62 (36.0)	
Gender Diverse	55 (5.7)	38 (4.8)	17 (9.9)	
**Sexual orientation**				**0.004**
Heterosexual	663 (69.3)	560 (71.3)	103 (60.2)	
Homosexual	75 (7.8)	53 (6.7)	22 (12.9)	
Bisexual	146 (15.3)	111 (14.1)	35 (20.5)	
Other or questioning	73 (7.6)	62 (7.9)	11 (6.4)	
**Age, mean (SD)**	21.69 (5.04)	21.63 (5.03)	21.99 (5.08)	0.389
**Foster youth**				**0.001**
Yes	61 (6.5)	37 (4.9)	24 (14.0)	
No	874 (93.5)	726 (95.1)	148 (86.0)	
**Financial stability**				**0.008**
Not stable at all	104 (11.3)	89 (11.8)	15 (8.8)	
Slightly unstable	327 (35.4)	267 (35.5)	60 (35.1)	
Stable	356 (38.6)	299 (39.8)	57 (33.3)	
Very Stable	136 (14.7)	97 (12.9)	39 (22.8)	
**Wondered how to obtain next meal**				**0.001**
Never wonder	439 (45.2)	382 (47.8)	57 (33.1)	
Rarely wonder	219 (22.6)	182 (22.8)	37 (21.5)	
Occasionally wonder	179 (18.4)	152 (19.0)	27 (15.7)	
Often wonder	91 (9.4)	56 (7.0)	35 (20.3)	
Always wonder	43 (4.4)	27 (3.4)	16 (9.3)	
**Illicit substance use while in college**				**0.001**
Yes, knowingly	472 (51.5)	372 (50.0)	100 (58.1)	
Yes, unknowingly	71 (7.8)	40 (5.4)	31 (18.0)	
No	373 (40.7)	332 (44.6)	41 (23.8)	
Exposure and campus context				
**Knew a student who sold sex or was trafficked**				**0.001**
Yes	232 (23.9)	146 (18.3)	86 (50.0)	
No	739 (76.1)	653 (81.7)	86 (50.0)	
**Nature of the sexual exchange** (n = 454)**				**0.012**
Forced	56 (12.3)	45 (14.2)	11 (8.1)	
Coerced	124 (27.3)	79 (24.8)	45 (33.1)	
Deceived	112 (24.7)	72 (22.6)	40 (29.4)	
Threatened	59 (13.0)	39 (12.3)	20 (14.7)	
Not externally influenced	79 (17.4)	61 (19.2)	18 (13.2)	
Other	24 (5.3)	22 (6.9)	2 (1.5)	
**Heard, saw, or suspected trafficking recruitment on campus**				**0.001**
Yes	107 (12.5)	73 (10.6)	34 (20.1)	
No or unsure	749 (87.5)	614 (89.4)	135 (79.9)	
**Ever sold sex as a college student**				**0.001**
Sold sex one or more times	178 (21.8)	99 (15.6)	79 (47.9)	
Considered it	168 (20.6)	136 (20.9)	32 (19.4)	
Never	469 (57.6)	415 (63.8)	54 (32.7)	
**Reason for sex exchange**				
To financially provide for someone else	191 (19.7)	148 (18.5)	43 (25.0)	**.054**
To have extra cash for spending	256 (26.4)	191 (23.9)	65 (37.8)	**.001**
To pay for school	246 (25.3)	178 (22.3)	68 (39.5)	**.001**
To pay for rent	223 (23.0)	164 (20.5)	59 (34.3)	**.001**
To pay for food	225 (23.2)	163 (20.4)	62 (36.1)	**.001**
**Exchanged sex across the US-Mexico border as a college student (self or other)**				**0.001**
Yes	135 (16.3)	75 (11.3)	60 (35.9)	
No	693 (83.7)	586 (88.7)	107 (64.1)	
Experiences arising from exchanging sex (n=626)**				
Physical abuse or violence	185 (29.6)	116 (24.2)	69 (47.3)	**0.001**
Emotional or psychological harm	267 (42.7)	180 (37.5)	87 (59.6)	**0.001**
Rape, sexual assault, sexual violence	154 (24.6)	105 (21.9)	49 (33.6)	**0.004**
Told or asked to recruit others	102 (16.3)	62 (12.9)	40 (27.4)	**0.001**
**STI**				**0.001**
Yes	140 (14.4)	87 (10.9)	53 (30.8)	
No	831 (85.6)	712 (89.1)	119 (69.2)	
**Felt pressure to engage in sexual activity by others in college**				**0.001**
Yes	202 (20.8)	119 (14.9)	83 (48.3)	
No	769 (79.2)	680 (85.1)	89 (51.7)	
Source of pressure to engage in sex (n = 194)**				0.747
Instructor	43 (22.2)	22 (19.8)	21 (25.3)	
Student	109 (56.2)	63 (56.8)	46 (55.4)	
Partner	33 (17.0)	21 (18.9)	12 (14.5)	
Other or Unsure	9 (4.6)	5 (4.5)	4 (4.8)	
**Felt pressure to transport or sell drugs (by someone they met from being on campus)**				**0.001**
Yes	46 (4.7)	27 (3.4)	19 (11.0)	
No	925 (95.3)	772 (96.6)	153 (89.0)	
**Felt pressure to perform labor**				**0.001**
Yes	33 (3.4)	19 (2.4)	14 (8.1)	
No	938 (96.6)	780 (97.6)	158 (91.9)	
**Felt pressure to exchange sex for help with schoolwork or grades**				**0.001**
Yes	47 (4.8)	27 (3.4)	20 (11.6)	
No	924 (95.2)	772 (96.6)	152 (88.4)	
**Greek fraternity/sorority organization affiliation**				**0.011**
Greek affiliated	115 (12.0)	85 (10.8)	30 (17.9)	
Not affiliated	841 (88.0)	703 (89.2)	138 (82.1)	
**Exchanging sex inhibited you from help-seeking the following on campus**				
Police	100 (10.3)	67 (8.4)	33 (19.2)	**0.001**
Mental health services	181 (18.6)	117 (14.6)	64 (37.2)	**0.001**
Academic counseling	130 (13.4)	73 (9.1)	57 (33.1)	**0.001**
Health services	141 (14.5)	90 (11.3)	51 (29.7)	**0.001**
Food services	50 (5.2)	35 (4.4)	15 (8.7)	**0.019**
Housing services	71 (7.3)	39 (4.9)	32 (18.6)	**0.001**
**Barriers to help-seeking on campus**				
Shame	221 (22.3)	149 (19.2)	62 (36.9)	**0.001**
Fear	222 (23.5)	153 (19.7)	69 (41.1)	**0.001**
Stigma	207 (21.9)	137 (17.6)	70 (41.7)	**0.001**
Discrimination	142 (15.0)	85 (10.9)	57 (33.9)	**0.001**
Didn’t want family or friends to know	186 (19.7)	119 (15.3)	67 (39.9)	**0.001**
Felt threatened by person facilitating sex	95 (10.0)	51 (6.6)	44 (26.2)	**0.001**
Didn’t want to expose the experience	84 (8.9)	50 (6.4)	34 (20.2)	**0.001**
Cost	40 (4.2)	24 (3.1)	16 (9.5)	**0.001**
Transportation	33 (3.5)	18 (2.3)	15 (8.9)	**0.001**
Unaware of where to get help	72 (7.6)	27 (16.1)	27 (16.1)	**0.001**
Insurance	37 (3.9)	8 (4.8)	8 (4.8)	0.531
Other	14 (1.5)	6 (3.6)	6 (3.6)	**0.013**
**Recommendations for addressing human trafficking on campus**				
More counselors with exploitation experience	588 (64.3)	472 (63.4)	116 (67.8)	0.279
More culturally, racially, and ethnically similar counselors	481 (52.6)	375 (50.4)	106 (62.0)	**0.006**
Support groups and organizations	536 (58.6)	414 (55.7)	122 (71.4)	**0.001**
Anonymous way to report	540 (59.0)	439 (59.0)	101 (59.1)	0.989
Improved campus security	377 (41.2)	296 (39.8)	81 (47.4)	0.069
More consequences for those who exploit	455 (49.7)	372 (49.9)	83 (48.5)	0.742
Human trafficking education on campus	456 (49.8)	365 (49.1)	91 (53.2)	0.327
Remove consequences for seeking help	373 (40.7)	320 (43.0)	53 (31.0)	**0.004**
Other	8 (0.9)	5 (0.7)	3 (1.8)	0.170

*Due to missing data, certain percentages may reflect smaller N than the column head or a larger N for multiple select answers. ** Excluding those who said “Not applicable” or “Prefer not to respond”. The p-values significant at the p<.05 level are in bold, indicating a statistically significant difference.

Multiple logistic regressions explored the factors significantly associated with being human trafficked as a college student. Models were developed using a manual procedure where all variables that attained a significance level <10% in univariate models were considered in multivariate analyses in order of most to least significance, and some interactions were explored. To address potential collinearity among variables, the variables were put in the regression model one at a time, using a forward stepwise approach, and only retained if they remained statistically significant at the p<.05 level.

## Results

College students (n = 971) from 12 campuses in Southern California, primarily located in the San Diego and Imperial Valley regions, completed the surveys. However, students also described multiple affiliations, with some having spent time at 35 other California colleges/universities throughout the state and in Arizona (23). Participants were predominantly undergraduate (86%), female (69%), male (25%), and gender diverse individuals (6%). Two-thirds (66%) reported their sexual orientation as heterosexual; 15% identified as bisexual, 8% as gay/lesbian, and 8% as other. They were 38% White, 36% Latino/a, 18% Asian/Pacific Islander, 17% Black, 5% American Indian/Native Alaskan, and 2% “other.” Sixty-one (7%) were former foster youth (**Table 1**).

Nearly one in five students (18%) who answered the surveys reported having experienced human trafficking (force, coercion, fraud into selling sex) during college (77% were undergraduate students); 13% heard, saw, or suspected trafficking recruitment occurring on campus. **Table 1** provides the reasons participants said they exchanged sex for something of monetary or other value (e.g,. for school, rent, food, or to support others). Further, many students reported having known someone who sold sex or was trafficked as a college student (24%) and described the nature of their peers’ sexual exchanges as having been coerced (27%), deceived (25%), threatened (13%), and forced (12%) into commercial sex exchanges. Students also reported traveling across the border to sell sex (or knowing students who did so) as college students (16%). They reported how commercial sex involvement (trafficked or not, self or other as a college student) impacted student safety and well-being. As a result, they experienced physical abuse or violence (30%). Others experienced emotional or psychological harm (43%), rape, sexual assault, or other sexual violence (25%), and being told or asked to recruit others to sell sex (16%).

Many students described feeling pressure by others to engage in sexual activities during college (21%). Students described pressure to engage in sex as a college student as originating from another student (44%), romantic partners (23%), professors (15%), and other individuals (9%). Students also described their intentions to sell sex, with 20% having considered it, 21% selling sex at least once, and 55% never considering it.

Bivariate analyses in **Table 1** show the following significant associations with being trafficked as a college student as more likely to: be BIPOC, LGBTQ, a foster youth or former foster youth, use illicit substances, be coerced and deceived into exchanging sex, have higher abuse of all types (physical, emotional, sexual, and being told to recruit others) during commercial sex exchanges, and more likely to have pressure to engage in sexual activity in college, transport/sell drugs, perform labor, and exchange sex for grades/schoolwork. If they were trafficked as a college student, they were more likely to be affiliated with a Greek fraternity/sorority. They were also more likely to be inhibited to seek help on campus from the police, mental health services, academic counseling, health services, food services, and housing services. Graduate students were more likely to be trafficked sometime as a college student than not, though they represented a smaller number of respondents overall. While 14% of students surveyed had acquired a sexually transmitted infection (STI) as a college student, 31% of those trafficked had an STI.

**Table 1** displays how students experienced shame, fear, and stigma as the top barriers to help-seeking on campus. Twenty-six percent of trafficked students said a barrier to help-seeking on campus was feeling threatened by the person facilitating the sex exchange. Specific recommendations included increasing the number of counselors with experience in exploitation and from culturally similar backgrounds. Students wanted support groups and the removal of consequences for seeking help. Half expressed the need for human trafficking education on campus and for more consequences for those who exploit. Survivors, such as community advisory board members, said that interventions during their first year as college students would have really helped.

**Table 2** illustrates the significant factors independently associated with being trafficked as a college student (n=970, with one variable missing data) when adjusting for age, sexual orientation, race, and the other factors retained in the model. Those who were trafficked (forced/coerced) into exchanging sex for something of monetary or other value were four times more likely to have exchanged sex as college students across the U.S.-Mexico border (OR = 4.02; CI: 2.52-6.17); nearly three times more likely to be inhibited to seek academic counseling (OR = 2.58; CI = 1.63-4.10); more likely acquire a sexually transmitted infection as a college student (reporting for self or other they knew who exchanged sex as a college student) (OR:1.63; CI: 1.04-2.57); more likely to wonder how they would afford the next meal (OR:1.20; CI: 1.03-1.40); and more than twice as likely to feel pressure from someone to engage in sex during college (OR:2.69; CI:11.75-4.12). Other factors, aside from the socio-demographic covariates, were no longer significant in the adjusted models and therefore not retained.

**Table 2:** Factors independently associated with being trafficked as a college student, respondents across 12 San Diego County/Imperial Valley colleges/universities, multiple logistic regression* (n=970).

Variable	Adjusted OR	Odds ratio 95% CI
Exchanged sex as a college student across the U.S.-Mexico border (self or other student)	4.02	2.52-6.17
Exchanging sex Inhibited them from seeking academic counseling	2.58	1.63-4.10
Acquired a sexually transmitted infection as a college student (if self or other student they knew exchanged sex)	1.63	1.04-2.57
Wondered how they would afford next meal	1.20	1.03-1.40
Felt pressured by others on campus to engage in sexual activity	2.69	11.75-4.12

*Adjusting for age, sexual orientation, and race.

## Discussion

This study found that one in five U.S. college students (of 971 surveyed across 12 campuses in southern California) experienced human trafficking during college. A recent student thesis at a Virginia campus found a similar proportion of students trafficked during college in their sample (24). In the current study, we found that human trafficking occurred across a range of university types: four-year public and private universities and within community college districts, without exception. Our findings suggest that traffickers have identified college campuses and other places where college students congregate (e.g., parties, malls, clubs) as prime geographical recruitment targets. The results represent an alarming reminder that the immense profitability of trafficking leads to continuously adaptive methods to lure victims. College campuses are no exception, yet the present study is one of the only ones to survey U.S. college students about their direct experiences with human trafficking and sexual exploitation in college.

In bivariate analyses, college students were more likely to be trafficked (coerced, forced, or frauded) than not when they were Black, Latine, male, LGBT, foster care system involved, or part of a fraternity or sorority. All of these populations tend to be understudied in the human trafficking literature. Other factors significantly associated with being sex trafficked were substance use, and the pressure to perform labor, transport or sell drugs, and exchange sex for help with grades and schoolwork. In addition, over three-fourths of those trafficked were undergraduate students. However, a higher percent of graduate students reporting that they were trafficked than not might be due to the fact they were in school longer than freshmen and therefore, experienced it more. When controlling for these characteristics, however, other factors emerged as more statistically significantly associated with being trafficked, as follows.

Students were four times more likely to be trafficked as college students across the U.S.- Mexico border. Studies involving women in Tijuana’s commercial sex industry have documented the incidence of trafficking and sexual exploitation among minors and adults, as well as their potential to organize against violence (25, 26). However, few studies have examined the heightened risk of human trafficking for U.S. college students hear the U.S.-Mexico border. Further research is needed to inform prevention strategies tailored to college students who experience cross-border trafficking.

Students who reported being trafficked in college also had a two-fold increase in the risk of acquiring a sexually transmitted infection (STI) as a college student, compared to those who were not trafficked, when controlling for other factors. This disparity is especially concerning given that students who experienced trafficking also reported barriers which prevented them from seeking support on campus, including health services. Further, that the elevated risk of STI among trafficked populations of students remained significant after controlling for sexual orientation and race and ethnicity, is particularly notable given that LGBTQ and BIPOC young adults already face an elevated risk of STI in general populations (27) and were inhibited to seek services on campus, such as health services, if they were trafficked.

Students were also more likely to be trafficked if they wondered how they would afford their next meal. Studies have shown the high incidence of food and housing insecurity among U.S. college students in recent decades (28, 29). Many U.S. universities and colleges, including those in San Diego and southern California where this study took place, have created campus Basic Needs Centers, Economic Crisis Response Teams, and housing specifically for unhoused students. Yet in this study, students were inhibited from seeking mental health, food, and housing services when they were trafficked as college students.

However, when controlling for other factors, students who were trafficked vs. not trafficked were especially more likely to be inhibited to seek academic counseling compared to other services. This finding is especially relevant to the goal of universities and colleges to educate students. Also, students were more likely to be pressured by someone (i.e. instructors, peers, partners) to engage in sex during college. Studies have documented sexual assault of college students (30, 31), but fewer studies have focused on the exploitation of college students in “quid pro quo” sex exchanges, nor framed them as commercial sexual exploitation or human trafficking, until now.

The study’s results should be interpreted with caution because not all students were surveyed as it was not a randomized sample, nor was it a survey of all students on all campuses. Future studies could add human trafficking/sexual exploitation questions to surveys that are routinely administered to all students on college campuses, particularly undergraduates to estimate the actual prevalence of human trafficking/sexual exploitation on a campus. As in most surveys, students self-selected to take the present survey. Thus, the large number of respondents who reported trafficking and sexual exploitative experiences suggests that these experiences occur among college students to a larger extent than the public may expect.

### Policy implications

Under U.S. legislation, Title IX, colleges and universities address sexual assault on campuses (32). Under Title IX, human trafficking or commercial sexual exploitation falls under the broader categories of sexual violence, sexual harassment, and sexual assault. They are not uniformly addressed across campuses. Therefore, the state of Virginia recently enacted legislation mandating human trafficking information in first-year orientations (33). However, formal evaluations are lacking on the effectiveness of such campaigns for college students. More research on the risk factors leading to commercial sexual exploitation among college students, and the nuances that lead to students’ exit from exploitation, are needed.

Other articles have noted the need for more data to be gathered to support the implementation of Title IX measures that would focus on human trafficking awareness (34). A 2025 California Violence Experiences (CalVEX) Survey also found adults with some college education as more likely to have experienced sexual exploitation than adults without any college education or those with postgraduate training and they were three times more likely to have experienced it when they had lower income (35). Another article described measuring human trafficking awareness on college campuses before and after U.S. college students received information about it (36). More studies are needed to test, implement, and evaluate human trafficking interventions on college campuses.

Recommendations to curb this sexual exploitation crisis include expanding 1) Title IX implementation to encompass human trafficking/sexual exploitation information at colleges/universities, particularly in new student orientations, and 2) state laws mandating human trafficking awareness training at middle/high schools to include colleges/universities. Strategies to prevent human trafficking among college students could target incoming student orientations throughout their first year. Strategies could entail removing consequences for students who seek help, and increasing campus support groups and counselors who understand them. Training of college personnel (e.g., counselors, student affairs officers, human resource personnel, university lawyers faculty) could also help detect and assist students experiencing sexual exploitation, including those living in dorms away from their parents/caregivers.

## Data Availability

The raw data supporting the conclusions of this article will be made available by the authors, without undue reservation. For access to the raw data or the survey instrument, contact Dr. Lianne A. Urada, the Principal Investigator, at lurada@sdsu.edu

## References

[1] Nemeth JM Rizo CF . Estimating the prevalence of human trafficking: Progress made and future directions. Am J Public Health. (2019) 109:1318–9. doi: 10.2105/AJPH.2019.305258. PMID: 31483724 PMC6727304

[2] National Human Trafficking Hotline . National Human Trafficking Hotline. Available online at: https://humantraffickinghotline.org/en/statistics/. (Accessed April 1, 2026).

[3] U.S. Congress . Victims of Trafficking and Violence Protection Act of 2000. Public Law 106-386, 114 Stat. 1464. (2000). Washington, D.C.: GPO.

[4] Anderson JE Cheney R Clatts M Faruque S Kipke M Long A . HIV risk behavior, street outreach, and condom use in eight high-risk populations. AIDS Educ Prev. (1996) 8:191–204. doi: 10.1521/aeap.1996.8.3.191. PMID: 8806949

[5] Carpenter A Gates J . The nature and extent of gang involvement in sex trafficking in San Diego County. Washington, DC: United States Department of Justice (2016). Available online at: https://cdn.kpbs.or6g/news/documents/2017/05/18/traffickingreport.pdf. (Accessed April 1, 2026).

[6] Cole J Sprang G Lee R Cohen J . The trauma of commercial sexual exploitation of youth: A comparison of CSE victims to sexual abuse victims in a clinical sample. J Interpersonal Violence. (2016) 31:122–46. doi: 10.1177/0886260514555133. PMID: 25381275

[7] Rothman EF Bazzi AR Bair-Merritt M . I’ll do whatever as long as you keep telling me that I’m important: A case study illustrating the link between adolescent dating violence and sex trafficking victimization. J Appl Res Children. (2015) 6:1–23. doi: 10.1027/00419-000. PMID: 31409215

[8] Ulloa E Salazar M Monjaras L . Prevalence and correlates of sex exchange among a nationally representative sample of adolescents and young adults. J Child Sex Abus. (2016) 25:524–37. doi: 10.1080/10538712.2016.1167802. PMID: 27266400 PMC5613935

[9] U.S. Department of Health Services, Blue Campaign . Human trafficking awareness guide for student leaders on college campuses. Available online at: https://www.dhs.gov/sites/default/files/2025-06/25_0605_bc_student-leaders-toolkit-v03-508.pdf. (Accessed April 1, 2026).

[10] Steinberg L . Adolescence (7th ed.). New York: McGraw-Hill, (2009).

[11] Drake EC Sladek MR Doane LD . Daily cortisol activity, loneliness, and coping efficacy in late adolescence: A longitudinal study of the transition to college. Int J Behav Dev. (2016) 40:334–45. doi: 10.1177/0165025415581914. PMID: 28979055 PMC5624735

[12] Crutchfield RM Maguire J Campbell CD Lohay D Valverde Loscko S Simon R . I’m supposed to be helping others”: Exploring food insecurity and homelessness for social work students. J Soc Work Educ. (2020) 56:S150–62. doi: 10.1080/10437797.2020.1741478. PMID: 41858497

[13] Cameron M Johnson R Lacy TA Wu J Siegel P Holley J . 2019–20 National Postsecondary Student Aid Study (NPSAS:20): First look at student financial aid estimates for 2019–20 (NCES 2023–466). In: U.S. Department of Education, National Center for Education Statistics Washington, DC: Department of Education, National Center for Education Statistics. (2023). Available online at: https://nces.ed.gov/pubsearch/pubsinfo.asp?pubid=2023466. (Accessed April 1, 2026).

[14] California State Legislature, Assembly . Assembly Bill 1227, 2017–2018 Regular Session: Human Trafficking Prevention Education and Training Act. Sacramento, CA: California State Legislature, 2017. California Legislative Information. (2017). Available online at: https://legiscan.com/CA/bill/AB1227/2017. (Accessed April 1, 2026).

[15] Rock A . Human trafficking on college campuses: What it looks like and resources for police, students. In: Campus Safety Magazine New York: Campus Safety Magazine, (2024). Available online at: https://www.campussafetymagazine.com/podcast/human-trafficking-college-campuses/. (Accessed April 1, 2026).

[16] Polaris . National Human Trafficking Hotline. 2019 Data Report. The U.S. National Human Trafficking Hotline. More victims and survivors speaking for themselves (2019). Available online at: https://humantraffickinghotline.org/sites/default/files/2022-12/Polaris-2019-US-National-Human-Trafficking-Hotline-Data-Report.pdf. (Accessed April 1, 2026).

[17] Goldenberg SM Rangel G Staines H Vera A Lozada R Nguyen L . Individual, interpersonal, and social-structural correlates of involuntary sex exchange among female sex workers in two Mexico-U.S. border cities. J Acquir Immune Defic Syndr. (2013) 63:639–46. doi: 10.1097/QAI.0b013e318296de71. PMID: 23614997 PMC3805673

[18] Gonzalez C Brouwer KC Reed E Nicholls MJ Kim J Gonzalez-Zuniga PE . Women trading sex in a U.S.-Mexico border city: A qualitative study of the barriers and facilitators to finding community and voice. Sexes. (2020) 1:1–18. doi: 10.3390/sexes1010001. PMID: 34386640 PMC8357315

[19] Betzler F Köhler S Schlemm L . Sex work among students of higher education: A survey-based, cross-sectional study. Arch Sex Behav. (2015) 44:525–8. doi: 10.1007/s10508-014-0476-y. PMID: 25617011

[20] Sagar T Jones D Symons K Tyrie J Roberts R . Student involvement in the UK sex industry: Motivations and experiences. Br J Sociology. (2016) 67:697–718. doi: 10.1111/1468-4446.12216. PMID: 27643817

[21] The impact of event scale - revised (IES-R). Available online at: https://member.aanlcp.org/wp-content/uploads/2021/08/The-impact-of-event-scale.pdf. (Accessed April 1, 2026).

[22] The psychological sense of school membership scale by Goodman 1993. Available online at: https://youthrex.com/wp-content/uploads/2019/10/PSSM-Scale.pdf. (Accessed April 1, 2026).

[23] Urada LA . Nemeth program in human trafficking research at San Diego State University: Final Report (2023). Available online at: https://drive.google.com/file/d/1hvIb8eDyisoQm9HhYDmycu2MLOd-43JD/view?usp=sharing. (Accessed April 1, 2026).

[24] Lefeavers S . A higher education response to exploitation on campus. (Master’s thesis). Richmond: Virginia Commonwealth University. (2025). Available online at: https://scholarscompass.vcu.edu/etd/8051/. (Accessed April 1, 2026).

[25] Goldenberg SM Silverman JG Engstrom D Bojorquez-Chapela I Usita P Rolón ML . Exploring the context of trafficking and adolescent sex industry involvement in Tijuana, Mexico: Consequences for HIV risk and prevention. Violence Against Women. (2015) 21:478–99. doi: 10.1177/1077801215569079. PMID: 25648946 PMC4703414

[26] Urada LA Gaeta-Rivera A Kim J Gonzalez-Zuniga PE Brouwer KC . Mujeres Unidas: Addressing substance use, violence, and HIV risk through asset-based community development for women in the sex trade. Int J Environ Res Public Health. (2021) 18:3884. doi: 10.3390/ijerph18083884. PMID: 33917190 PMC8068011

[27] Centers for Disease Control and Prevention . Sexually transmitted disease surveillance 2021 (Archived Report). In: U.S. Department of Health and Human Services Atlanta: U.S. Department of Health and Human Services. (2024). Available online at: https://www.cdc.gov/sti-statistics/media/pdfs/2024/07/2021-STD-Surveillance-Report-PDF_ARCHIVED-2-16-24.pdf. (Accessed April 1, 2026).

[28] Freudenberg N Crutchfield R . Finding solutions: Meeting essential needs to overcome health and educational inequities among college students. Soc Sci. (2025) 14:654. doi: 10.3390/socsci14110654. PMID: 41725453

[29] Goldman BJ Freiria CN Landry MJ Arikawa AY Wright L . Research trends and gaps concerning food insecurity in college students in the United States: A scoping review. J Am Coll Health. (2025) 73:2960–99. doi: 10.1080/07448481.2024.2351420. PMID: 38870038

[30] Fedina L Holmes JL Backes BL . Campus sexual assault: A systematic review of prevalence research from 2000 to 2015. Trauma Violence Abuse. (2018) 19:76–93. doi: 10.1177/1524838016631129. PMID: 26906086

[31] Wagman JA Amabile C Sumstine S Park E Boyce S Silverman J . Student, staff, and faculty perspectives on intimate partner and sexual violence on 3 public university campuses: Protocol for the UC Speaks Up Study and preliminary results. JMIR Res Protoc. (2022) 11:e31189. doi: 10.2196/31189. PMID: 35380114 PMC9019617

[32] United States Congress . Title IX of the Education Amendments of 1972. Washington, DC: U.S. Government Printing Office. (1972).

[33] Virginia General Assembly . (2023). Va. Code § 23.1-808.1. Human trafficking awareness and prevention training; first-year orientation. Code of Virginia. Richmond: Commonwealth of Virginia. Available online at: https://law.lis.virginia.gov/vacode/title23.1/chapter8/section23.1-808.1/ (Accessed April 1, 2026).

[34] Preble KM Cook MA Fults B . Sex trafficking and the role of institutions of higher education: Recommendations for response and preparedness. Innov High Educ. (2019) 44:5–19. doi: 10.1007/s10755-018-9443-1. PMID: 41873353

[35] Urada L Patel P Rao N Kully G Thomas J Raj A . (2026). Sexual Exploitation in California: Findings from CalVEX 2025. Violence Experiences Study (VEX). University of California San Diego and the Newcomb Institute at Tulane University.

[36] Hill K Calk PT Bradley E Hunt RE Lauer MC Lee DR . Implementation of a human trafficking educational module for college students: Pre/post design. Open J Occup Ther. (2023) 11:1–7. doi: 10.15453/2168-6408.2009

[37] Urada LA Weitensteiner A . Human trafficking and sexual exploitation at U.S. colleges/universities: Policy implications to halt it. medRxiv [Preprint]. (2025) doi: 10.64898/2025.12.31.25342184. PMID: 41510840

